# Emergence of ceftazidime-avibactam-resistant *Klebsiella pneumoniae* during treatment, Finland, December 2018

**DOI:** 10.2807/1560-7917.ES.2019.24.19.1900256

**Published:** 2019-05-09

**Authors:** Kati Räisänen, Irma Koivula, Heikki Ilmavirta, Santeri Puranen, Teemu Kallonen, Outi Lyytikäinen, Jari Jalava

**Affiliations:** 1 Department of Health Security, National Institute for Health and Welfare, Helsinki, Finland; 2 Kuopio University Hospital, Unit of Infections and Hospital hygiene, Kuopio University Hospital, Kuopio, Finland; 3 Eastern Finland laboratory Centre, Kuopio, Finland; 4 Aalto University, Department of Computer Science, Espoo, Finland; 5 Department of Biostatistics, University of Oslo, Oslo, Norway

**Keywords:** *Klebsiella pneumoniae*, carbapenem, ceftazidime-avibactam, resistance, whole-genome sequencing, KPC-2

## Abstract

In December 2018, a ceftazidime-avibactam (CAZ-AVI)-resistant KPC-2-producing *Klebsiella pneumoniae* strain was isolated in Finland. CAZ-AVI resistance was observed 34 days after CAZ-AVI treatment in a trauma patient transferred from a hospital in Greece who had been colonised with bla_KPC-2_-producing *K. pneumoniae* ST39, and later developed a bloodstream infection. The CAZ-AVI-resistant strain contained a novel 15 amino acid insertion in the KPC-2 protein causing structural changes proximal to the KPC-2 active site.

Ceftazidime-avibactam (CAZ-AVI) is a promising novel β-lactam-β-lactamase inhibitor combination with activity against multidrug-resistant (MDR) Enterobacteriaceae for which there are limited treatment options, e.g. those producing *Klebsiella pneumoniae* carbapenemases (KPC). According to the rapid risk assessment published by the European Centre for Disease Prevention and Control (ECDC) in June 2018, the emergence of CAZ-AVI resistance in Europe is a very rare phenomenon but an important cross-border threat that should be monitored carefully [1]. Here, we report the emergence of CAZ-AVI resistance in a KPC-2-producing *K. pneumoniae* strain and describe a mutation associated with the resistance.

## Case description

Following a traffic accident, a patient without any known underlying diseases was hospitalised in Greece. The patient was first admitted to a local hospital before being transferred to a tertiary hospital 3 days later; 27 days after admittance the patient was moved to a university hospital in Finland. There was no information on carriage of carbapenemase-producing Enterobacteriaceae (CPE) in the medical notes received from the Greek hospital. The screening specimens for MDR bacteria were obtained at admission to the Finnish hospital according to the national guidelines [2] including rectal swabs and skin lesions. The case was isolated and treated with contact precautions. The antimicrobials (metronidazole, vancomycin, tigecycline and colistin) that had been prescribed in Greece were stopped after admittance since the patient showed no clinical signs or symptoms of infection.

The screening specimen from sacrum decubitus was positive for KPC-2-producing *K. pneumoniae* sequence type (ST)39 (isolate 1), which was susceptible to CAZ-AVI and resistant to carbapenems and several other antimicrobials (Table). The patient developed fever 16 days after admission to the hospital in Finland. Treatment with tigecyclin and CAZ-AVI was initiated after obtaining blood cultures, which yielded KPC-2-producing *K. pneumoniae* ST39 (isolate 2), susceptible to CAZ-AVI and resistant to carbapenems. The treatment with tigecyclin and CAZ-AVI was administered for 2 weeks. After 2 days without antimicrobials, the patient developed fever again and treatment with CAZ-AVI and fosfomycin was initiated and continued for 19 days. Ten days after treatment ended, the patient developed fever once more. Blood cultures were found to be positive for KPC-2-producing *K. pneumoniae* ST39 (isolate 3), resistant to both CAZ-AVI and carbapenems but sensitive to sulfamethoxazole-trimethoprim. Colistin and sulfamethoxazole-trimethoprim were administered for 12 days. The patient subsequently recovered from the infection.

**Table:** Antimicrobial susceptibility and genetic characteristics of *Klebsiella pneumoniae* clinical isolates, Finland, October–December 2018

Isolate	Type of specimens	MIC (µL/mL)							β-lactamase resistance genes
		ETP	MEM	CAZ-AVI	CST	FOF	SXT	TGC	
Isolate 1	Sacrum, decubitus	> 32	> 32	1	8	64	R^a^	1	bla_KPC-2_, bla_TEM-1_, bla_SHV-11_
Isolate 2	Blood	> 32	> 32	< 1	> 8	32	R^a^	4	bla_KPC-2_, bla_TEM-1_, bla_SHV-11_
Isolate 3	Blood	6	16	> 16	0,5	> 1,024	0,38	1,5	bla_KPC-2 variant_^b^, bla_SHV-11_

CAZ-AVI: Ceftazidime-avibactam; CST: colistin; ETP: ertapenem; FOF: fosfomycin; MEM, meropenem; MIC: minimum inhibitory concentration; SXT: trimethoprim-sulfamethoxazole; TGC: tigecycline.

^a ^Only interpretation results of susceptibility testing were available and strains were resistant (R).

^b ^15 amino acids insertion (AVYTRAPNKDDKHSE) of KPC-2 variant after position 259.

Susceptibility testing and interpretation were carried out according to the European Committee on Antimicrobial Susceptibility Testing breakpoints [12].

## Molecular characterisation of CAZ-AVI resistance mechanism

All three KPC-2-producing *K. pneumoniae* ST39 isolates were characterised by whole genome sequencing method and subsequent data by core-genome multilocus sequence typing (cgMLST) as previously described [3]. The isolated strains, with six to 13 allelic differences, were all categorised as belonging to the same clone according to the cgMLST [4]. The first two (isolates 1 and 2) had three β-lactam resistance genes (bla_TEM-1_, bla_SHV-11_ and bla_KPC-2_) and the third (isolate 3) had two such genes (bla_SHV-11_ and a bla_KPC-2_ variant). The third isolate had lower minimal inhibition concentrations (MIC) of meropenem and ertapenem than those of the first two isolates. The bla_KPC-2_ genes from the first two isolates were identical. The bla_KPC-2_ gene from the third isolate had an insertion of 45 nucleotide corresponding to 15 amino acid insertion after position 259 of the KPC-2 protein (GenBank accession number MK823188).

## Discussion

After the ECDC published its rapid risk assessment of the emergence of CAZ-AVI resistance, the National Institute for Health and Welfare (THL) informed clinical microbiology laboratories in Finland and asked them to notify THL of any CAZ-AVI resistant isolates. To our knowledge, this is the first CAZ-AVI-resistant *K. pneumoniae* with bla_KPC_ gene isolated in Finland and the second isolated in Europe.

CAZ-AVI resistance was observed after 34 days of CAZ-AVI treatment in a patient who was colonised by bla_KPC-2_-producing *K. pneumoniae* ST39 and later developed a blood stream infection. The strain isolated after CAZ-AVI treatment had a mutated bla_KPC-2_ gene encoding KPC-2 protein with 15 amino acid insertion; the observed mutation in bla_KPC-2_ gene has not been described previously. Comparative protein modelling [5,6] based an inhibitor-blocked KPC-2 crystal structure (PDB ID: 5UJ4) reveals that the 15 amino acid insertion adds to a loop region connecting the central beta-sheet to the carboxyl-terminal alpha-helix proximal to the KPC-2 active site.

We hypothesise that the resulting structural change weakens avibactam's inhibitory effect by disrupting its ability to bind at the active site, thereby causing resistance. Mutations in bla_KPC_ genes have been associated with the development of CAZ-AVI resistance after CAZ-AVI treatment in a few occasions. The first report from the United States (US) identified *K. pneumoniae* ST258 isolates with three mutations in KPC-3 (D179Y/T243M double substitution, D179Y and V240G) after 10–19 days of CAZ-AVI treatment [7,8]. The D179Y mutation in KPC-3 was also described in the *K. pneumoniae* ST1519 strain from Italy where CAZ-AVI resistance arose after 17 days of CAZ-AVI treatment [9]. The second report from the US described the same D179Y mutation in KPC-2 in *K. pneumoniae* ST258 isolate after 12 days of CAZ-AVI treatment [10]. In these earlier reports, KPC mutations causing CAZ-AVI resistance decreased meropenem MICs and some strains became even susceptible to meropenem. We detected similar decrease in meropenem MICs but our strain remained resistant. In addition to KPC gene mutations, CAZ-AVI resistance has been associated to CTX-M-14 mutations [11].

In conclusion, treating infections caused by KPC-producing *K. pneumonia* with CAZ-AVI can select KPC-variants that cause CAZ-AVI resistance and simultaneously preserve carbapenem resistance. Therefore, careful monitoring of resistance development by bacterial cultures and subsequent susceptibility testing is important.
